# High Organization-Based Self-Esteem Is Associated With Lower Risk of Depressive Symptoms Among University Athletes

**DOI:** 10.3389/fpsyg.2022.841271

**Published:** 2022-04-27

**Authors:** Ryusei Noguri, Yujiro Kawata, Shinji Yamaguchi, Nobuto Shibata, Tsuneyoshi Ota

**Affiliations:** ^1^Graduate School of Health and Sports Science, Juntendo University, Chiba, Japan; ^2^Institute of Health and Sports Science and Medicine, Juntendo University, Chiba, Japan; ^3^Faculty of Health and Sports Science, Juntendo University, Chiba, Japan; ^4^Juntendo Tokyo Koto Medical Center for the Elderly, Tokyo, Japan

**Keywords:** organization-based self-esteem, university athlete, depressive symptoms, risk, mental health

## Abstract

As depressive symptoms can impair athletes’ healthy competitive life and lead to a decline in performance, it is necessary to identify and prevent these symptoms. Organization-based self-esteem is one of the factors that influence the mental health of the members of an organization. It has been found that employees with high organization-based self-esteem have good mental health. However, the relationship between organization-based self-esteem and mental health has not yet been investigated in athletes. Therefore, we aimed to develop an organization-based self-esteem scale for university athletes (Study I) and investigate the relationship between organization-based self-esteem and depressive symptoms (Study II). Study I included subsample A: 210 university athletes (average age 19.6 ± 0.64 years) and subsample B: 371 university athletes (average age 19.4 ± 0.90), who responded to the newly developed Organization-Based Self-Esteem Scale for University Athletes (OBSE-UA), the Rosenberg Self-Esteem Scale, and the Sports Commitment Scale. To confirm the reliability of the developed scale, 2 weeks later they responded to the OBSE-UA again. In Study II, the participants were 232 university athletes (average age 19.5 ± 1.10 years), who completed the OBSE-UA developed in Study I and the Self-Rating Depression Scale. In Study I, we extracted a one-factor structure with six items for the OBSE-UA using exploratory and confirmatory factor analysis. Sufficient validity and reliability were confirmed by examining the relationship between organization-based self-esteem and sports commitment scale and retest methods, respectively. In Study II, athletes with high organization-based self-esteem showed a 0.33 time lower risk of experiencing depressive symptoms, compared to athletes with low organization-based self-esteem.

## Introduction

It has been reported that 34% of athletes have symptoms of anxiety or depression (Gouttebarge et al., 2019). Experiencing depressive symptoms may lower performance (Newman et al., 2016; Lochbaum et al., 2021) and lead to injuries (Yang et al., 2014). Additionally, mental health issues also cause drop out among athletes (Isoard-Gautheur et al., 2016).

In a recent review, factors related to mental health problems, such as depressive symptoms, were classified into following four categories: “personal risk factors,” “personal protective factors,” “sport-environmental risk factors,” and “sport-environmental protective factors” (Kuettel and Larsen, 2020). For example, injury and overtraining are personal risk factors, and basic needs and carrier satisfaction are personal protective factors. Additionally, while trusting and mastery-oriented climate are sport-environmental protective factors, sport-specific stressors and lack of support from teammates and coaches are sport-environmental risk factors.

Self-competence within organizations may be a factor that is related to athletes experiencing depressive symptoms. Bullying and mistreatment within sport organizations have been reported to be triggers for depressive symptoms (Lebrun et al., 2018). Mental health is impaired when athletes are unable to find their role within the organization to which they belong (Ojio et al., 2021). In contrast, individuals’ perception of their coaches’ acceptance of their performance has been reported to a protective factor against depressive symptoms (Doherty et al., 2016). Thus, athletes who cannot perceive their competence in their team are likely to have depressive symptoms.

The relationship between an individual and their organization is called “organization-based self-esteem.” Organization-based self-esteem is defined as “the degree to which an individual believes him or herself to be capable, significant, and worthy as an organizational member” (Pierce et al., 1989). Research on organization-based self-esteem had been accumulated in the field of occupational health. Additionally, it has been reported that it is associated with depressive symptoms, well-being, and supervisors’ leadership and support (Ferris et al., 2009; Bowling et al., 2010; Matsuda and Ishikawa, 2012; Zhang and Liu, 2019). A survey of corporate employees found that people with low self-esteem had more depressive symptoms than those with high self-esteem (Bowling et al., 2010). Thus, research on self-esteem within an organization has been actively conducted mainly in the field of occupational health, for corporate employees.

The 10-item Organization-Based Self-Esteem Scale (OBSE)— which has been widely used in research on organization-based self-esteem—is a self-report measure developed by Pierce et al. (1989) to measure organization-based self-esteem among corporate employees. In 2011, Matsuda and colleagues developed the Japanese version of the OBSE (OBSE-J) for Japanese employees. The OBSE consists of items such as “I am needed at work” and “I have trust in myself at work.” However, because the manner in which the items are phrased is not suitable for measuring the condition of athletes within an organization the OBSE cannot be used to measure athletes’ self-esteem within an organization. To the best of our knowledge, a scale to measure organization-based self-esteem among athletes has not been developed yet.

University athletes, like the employees of a company, are exposed to evaluation within the team, and at some point, the significance of their existence in the team becomes unstable. While some athletes are assigned roles such as captains and managers, some belong to the team but cannot act as regular or one-armed forces, and therefore, might lack self-esteem. Thus, there are differences in the level of athletes’ self-esteem within the organization even in the same team.

Self-esteem within the organization plays an important role in the field of sports science, just as in the field of occupational health. Organization-based self-esteem may vary depending on the method of coaching and instruction, such as role assignment and rights transfer within the organization. In addition, if university athletes’ organization-based self-esteem can be measured, the actual state of their self-esteem, which has not been clarified so far, can be grasped in detail. This can also help advance research on organization-based self-esteem and provide a way for university athletes to lead a healthy competitive life.

According to Kuettel and Larsen’s (2020) study, organization-based self-esteem can be a personal proactive factor for mental health, since it may help people maintain their mental health. Thus, we hypothesized the following:

Hypothesis: Athletes with high organization-based self-esteem are less prone to experiencing depressive symptoms.

This study is considered necessary for the following reasons: There is limited research on the relationship between organization-based self-esteem and depressive symptoms among athletes. Furthermore, self-competence within a team (a protective factor against depressive symptoms among athletes) has only been examined in case studies (Doherty et al., 2016). Therefore, to clarify ways to treat depressive symptoms, it is necessary to suggest pragmatic protective factors. Additionally, clarifying the relationship between organization-based self-esteem and depressive symptoms among athletes may contribute to the development of personal-approach and coaching methods to help address the depressive symptoms.

### Purpose

The purpose of this study was to develop a university athletes version of the OBSE and verify its validity and reliability (Study I), and to examine the relationship between organization-based self-esteem and risk for depressive symptoms among university athletes (Study II).

## Study I

### Method

#### Participants

Study I, which aimed to examine validity and reliability of the OBSE among university athletes, comprised two subsamples (A and B). We used subsample A, which consisted of 210 university athletes (105 male, 105 female; M_age_: 19.6 ± 0.64 years; mean competitive years: 8.8 ± 4.4 years) who belonged to the competition-oriented sports club of the university, to conduct an exploratory factor analysis. In addition, we used subsample B, which consisted of 371 university athletes (172 male, 199 female; M_age_: 19.4 ± 0.9 years; mean competitive years: 9.9 ± 3.9 years) who belonged to the competition-oriented sports club of the university, to conduct a confirmatory factor analysis. Furthermore, 93 participants (63 male, 30 female; M_age_: 20.2 ± 0.37 years; mean competitive years: 10.2 ± 3.7 years), who were randomly selected from subsample A, were included in the re-survey at an interval of 2 weeks, the assess their sociodemographic information and the OBSE’s the reliability among university athletes.

#### Procedure

Study I was conducted with the approval of the Research Ethics Committee of the Faculty of Health and Sports Science, Juntendo University (approval number: 2020-95). The purpose of the study and the anonymity of the data were explained to the participants. They were also informed of their right to decline to participate at any time without any reason, even after consenting to participate. Only those who agreed to participate in the study were included. All the participants provided written informed consent. Participants were recruited after college classes. Answers were collected via an online survey. Participants completed the surveys in a quiet environment.

#### Measures

Study I included the following measures:

1. Sociodemographic information (name, sex, age, competitive events, competitive years, role in the team, and competition results).

2. Original items used to develop the Organization-Based Self-Esteem Scale for University Athletes.

To develop the scale, we revised the original items in the OBSE (Pierce et al., 1989) and the Japanese Version of the Organization-Based Self-Esteem Scale (OBSE-J) (Matsuda et al., 2011). For instance, items in OBSE (Pierce et al., 1989) included the word “around here” (e.g., I am trusted around here); however, we replaced items in this scale with the word “team” to target athletes (e.g., I can make a difference in my team) (see, Table 1). To maintain the content validity, each item was selected by the consensus of a university faculty member specializing in sports psychology, a faculty member specializing in health psychology, a faculty member specializing in mental health as a psychiatrist, and a graduate student specializing in sports psychology who had more than 10 years of active sports experience. For each item, measurements were taken using a 5-point Likert scale ranging from “strongly disagree” to “strongly agree.” The mean score of all items was the total scale score; the higher the score, the higher the self-esteem within the organization. Moreover, OBSE (Pierce et al., 1989) and OBSE-J (Matsuda et al., 2011) are a one-factor-structured measure.

3. Japanese version of the Rosenberg Self-Esteem Scale.

**TABLE 1:** List of items on the Organization-based self-esteem scale and the organization-based self-esteem scale for University athletes.

No.	Organization-Based Self-Esteem Scale (Pierce et al., 1989)	Organization-Based Self-Esteem Scale for University Athletes (Developed in this study)
1	I count around here	I am needed in my team
*2	I am trusted around here	I am trusted in my team
3	I am helpful around here	I am helpful in my team
4	I am taken seriously around here	I am taken seriously in my team
*5	There is faith in me around here	There is faith in me in my team
6	I can make a difference around here	I can make a difference in my team
*7	I am a valuable member of this workplace	I am a valuable member of this team
8	I am cooperative around here	I am cooperative in my team
9	I am efficient around here	I am efficient in my team
*10	I am an important member of this workplace	I am an important member of this team

Item 1 (I count around here) was changed into “I am needed in my team”, according to the suggestion from Matsuda et al. (2011) which made a Japanese version of the Organization-Based Self-Esteem Scale. This is because the emphasis upon the words “I count” is of a vernacular commonly spoken in the West without a translation that is common to the Japanese language. Thus, the word “need” was used instead of “count” in Matsuda et al. (2011) study. This study referred to the suggestion, resulting in “I am needed in my team.” Items with asterisk (*) were omitted from the final version.

To measure the criterion-related validity of the Organization-Based Self-Esteem Scale for University Athletes (OBSE-UA), we used the Japanese version of the Rosenberg Self-Esteem Scale (RSES-J; Mimura and Griffiths, 2007), referring to previous studies (Pierce et al., 1989; Matsuda et al., 2011). The RSES-J was translated from the original Rosenberg Self-Esteem Scale (Rosenberg, 1965) into Japanese, and its validity and reliability have been confirmed (Mimura and Griffiths, 2007). The RSES-J has two factors: a positive attitude (six items) and a negative attitude (four items). The items are rated on a 4-point Likert: “strongly disagree,” “disagree,” “agree,” and “strongly agree.” The average score of each factor was calculated; higher scores indicated higher self-esteem.

4. Japanese version of the Sports Commitment Scale.

To measure the criterion-related validity of the OBSE-UA, we used the Japanese version of the Sports Commitment Scale (SCS-J; Hagiwara and Isogai, 2014), referring to previous studies (Pierce et al., 1989; Matsuda et al., 2011). The SCS-J was translated from the original Sports Commitment Scale (Scanlan et al., 2003) into Japanese, and its validity and reliability have been confirmed (Hagiwara and Isogai, 2014). The SCS-J consists of six items measured on 5-point Likert scales from “not at all dedicated” to “very dedicated” and “not at all hard” to “very hard.” The total scale score was calculated; higher scores indicated a higher level of sports commitment.

#### Data Analysis

##### Item Analysis

The mean and standard deviation of each item were calculated, and the ceiling and floor effects and measurement accuracy were checked for each item in the OBSE-UA. To examine the discriminating power of each item in the OBSE-UA, the participants were classified into the top 50% and bottom 50% of the scale scores for the 10 items, and a good-poor analysis (G-P analysis) was performed. In addition, item-total correlation analysis (I–T analysis) was performed for all 10 items of the OBSE-UA.

##### Validity

###### Content Validity

To maintain content validity, each item was selected by the consensus of the following specialists: a university faculty member specializing in sports psychology, a faculty member specializing in health psychology, a faculty member specializing in mental health as a psychiatrist, and a graduate student specializing in sports psychology who had more than 10 years of active sports experience.

###### Sampling Validity

Prior to performing the exploratory factor analysis, the validity of the sample was confirmed using the Kaiser-Meyer-Olkin (KMO) and Bartlett’s (BS) analysis. A KMO value of 0.90 or higher is considered excellent, and 0.80 is considered very good (Kaiser, 1974). If the significance is confirmed in the BS analysis, sufficient compatibility in factor analysis can be confirmed (Strickland, 2003).

###### Construct Validity

To determine the factor structure of the OBSE-UA, an exploratory factor analysis (maximum likelihood method/Promax rotation) was performed based on a factor loading of 0.40 or more. Next, confirmatory factor analysis was performed to confirm the model goodness-of-fit index by goodness-of-fit index (GFI), adjusted goodness of fit index (AGFI), comparative fit index (CFI), Tucker-Lewis index (TLI), root mean square error of approximation (RMSEA), and standardized root mean square residual (SRMR).

###### Criterion Validity

To investigate the criterion validity of the OBSE-UA, Pearson’s correlation analyses were performed using the total score of the OBSE-UA, each factor score of the RSES-J, and total score of the SCS-J, referring to previous studies on the OBSE (Pierce et al., 1989) and OBSE-J (Matsuda et al., 2011).

##### Reliability

###### Internal Consistency

To investigate the internal consistency of the scale, Cronbach’s alpha coefficients and Omega coefficients were calculated.

###### Reproducibility

To examine the reproducibility of the scale, we measured the OBSE-UA at 2-week intervals. Then, we calculated the intraclass correlation coefficient (ICC 1.1) using the 2-time measurement data of the OBSE-UA, as measured at 2-week intervals. Next, the Bland-Altman analysis was performed to examine the degree of agreement between the two OBSE-UA measurements. In the Bland-Altman analysis, the 95% confidence interval of the difference between the two measurement scores was calculated, and if the 95% confidence interval did not cross 0, it was judged that there was a fixed bias. When the correlation coefficient of the score difference between the two measurements and the average value of the two measurements was significantly associated, a proportional error was judged.

The software IBM SPSS Statistics version 27.0 (IBM Corp, Armonk, NY, United States), AMOS 21 (IBM Corp, Armonk, NY, United States), and Microsoft Excel 2020 (Microsoft, Seattle, WA, United States) were used for these analyses. The significance level for this study was less than 5%.

### Results

#### Item Analysis

No ceiling or floor effects were observed in any of the items. Therefore, all 10 items were used in subsequent analyses.

As it was assumed that there is a correlation between the total OBSE-UA score and each item of the OBSE-UA, Pearson’s product-moment correlation was used for the I–T analysis. A positive correlation with the total score was confirmed at the 0.1% level for all 10 items (*r* = 0.69–0.90, *p* < 0.001).

In addition, participants with a high total score on the OBSE-UA were shown to have a high score for each item. Therefore, based on the median OBSE-UA score, the participants were classified into the “OBSE-UA high group” and “OBSE-UA low group,” and the item scores were compared using *t*-tests. Significant differences were confirmed at the 0.1% level for all 10 items.

#### Validity

##### Sampling Validity

Before performing factor analysis for the OBSE-UA, we examined the sample validity to examine whether the sample size was sufficient. As a result, KMO was above 0.90 and BS value was significant, confirming that the data were suitable for factor analysis (KMO = 0.933, BS = 1816.41, *p* < 0.001).

##### Construct Validity

To clarify the factor structure of the OBSE-UA, we performed an exploratory factor analysis on the 10 items that were included in the original draft of the OBSE-UA. The items were selected based on a load of 0.40 or more for all factors. As a result, 10 items per factor were confirmed.

**FIGURE 1:**
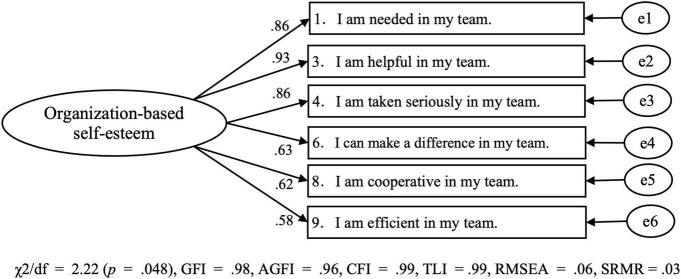
Confirmatory factor analysis for the Organization-Based Self-Esteem Scale for University Athletes.

Next, as a result of confirmatory factor analysis to confirm the model goodness of fit of the OBSE-UA, the path coefficients from the latent variable to the observed variable all satisfied the significance level. However, the model goodness-of-fit indicators were: χ^2^/*df* = 8.22 (*p* < 0.001), GFI = 0.86, AGFI = 0.78, CFI = 0.92, and RMSEA = 0.14, which were not statistically sufficient. Therefore, we checked the revised index, where values of 20 or more were shown in items 2, 5, 7, and 10. Based on this, it was concluded that these four items were redundant. Therefore, these items were excluded, and confirmatory factor analysis was performed again. As a result, a statistically sufficient model goodness of fit was obtained [χ^2^/df = 2.22 (*p* = 0.048), GFI = 0.98, AGFI = 0.96, CFI = 0.99, TLI = 0.99, RMSEA = 0.06, SRMR = 0.03]. Therefore, the final version of the OBSE-UA consisted of 6 items for the one factor (Figure 1).

##### Criterion Validity

To verify the criterion validity, we examined the relationship between the total score of the OBSE-UA and external criteria. The results showed that “positive attitude” (*r* = 0.51, *p* < 0.001) and “negative attitude” on the RSES-J (*r* = −0.28, *p* < 0.001), and total score on the SCS-J (*r* = 0.48, *p* < 0.001) had a significant correlation with the OBSE-UA.

#### Reliability

##### Internal Consistency

To examine the internal consistency of the OBSE-UA, we performed a reliability analysis using Cronbach’s alpha and Omega coefficient. The results showed a high internal consistency (α = 0.89, ω = 0.90) across the scale (Hayes and Coutts, 2020).

##### Reproducibility

To clarify the reproducibility of the OBSE-UA, we calculated the interclass correlation coefficient of the two measurements and found high reliability (ICC = 0.81, *p* < 0.001). Next, as a result of confirming the addition error and the proportionality error by the Bland-Altman analysis, neither fixed error (95% confidence interval: −0.15 to 0.13) nor proportionality error was observed (*r* = −0.04, *p* = 0.728).

## Study II

### Method

#### Participants

Study II was conducted between June to September 2021; participants were 232 athletes (average age: 19.5 ± 1.10 years; average competition history: 10.5 ± 3.50 years) who belonged to the competition-oriented sports club of the university.

#### Procedure

Study II was conducted with the approval of the Research Ethics Committee of the Faculty of Health and Sports Science, Juntendo University (approval number: 2020-95). The purpose of the study and the anonymity of the data were explained to the participants. They were also informed of their right to decline to participate at any time without any reason, even after consenting to participate. Only those who agreed to participate in the study were included. All the participants provided written informed consent. Participants were recruited after college classes. Answers were collected via an online survey. Participants completed the survey in a quiet environment.

#### Measures

The following measures were employed in the main study:

1. Demographic data (sex, role in the team, age, school year, competitive year, and competitive event).

2. Organization-Based Self-Esteem Scale for University Athletes (OBSE-UA).

The OBSE-UA was used to measure organization-based self-esteem in athletes. This scale revealed a one-factor structure with six items and showed good reliability and validity in Study I. Each item was rated on a 5-point Likert scale ranging from “strongly disagree” to “strongly agree.” A higher score indicated higher organization-based self-esteem.

3. Self-Rating Depression Scale.

The Self-Rating Depression Scale (SDS; Zung, 1965) was used to identify depressive symptoms in athletes. This is a reliable and valid tool, consisting of 20 items. The SDS items are scored on a scale from “1: not, occasionally” to “4: almost always.” The total score was calculated, with higher scores indicating more severe depressive symptoms. In this study, with reference to Zung (1973), 40–47 points were classified as mild depressive symptoms, 48–55 points as moderate, and 56 points or more as severe.

#### Data Analysis

##### Extracting Confounding Factors

To examine the confounding factors for depressive symptoms, we examined the relationship between individual attributes and depressive symptoms. Gender, role in the team, *t*-test for binary variables in the sport, age and grade, and sports history were analyzed by one-factor analysis of variance. Student’s *t*-test was performed for binary variables such as gender, role in the team, and one-factor analysis of variance was performed for multivalued variables such as age, grade, and competition history.

##### The Relationship Between Organization-Based Self-Esteem and Depressive Symptoms

Binomial logistic regression analysis was performed to examine the relationship between organization-based self-esteem and depressive symptoms among university athletes. The independent variable was based on the median OBSE-UA score (low group: less than three points, high group: three points or more), and the dependent variable was based on the cut-off value of SDS (low group: less than 48 points, high group: 48 points or more).

The SDS comprehensively captures 20 types of depressive symptoms. Therefore, to examine which depressive symptoms are associated with organization-based self-esteem, a binomial logistic regression analysis was also performed using the two groups based on the median OBSE-UA score and the midpoint of each symptom on the SDS; if the participant responded with “1: No, occasionally” or “2: Occasionally” to each item, they were included in the low group, whereas, if they responded with “3: For a long time” or “4: Almost always,” they were classified as the high group. IBM SPSS Statistics version 27.0 (IBM Corp, Armonk, NY, United States) was used for all statistical analyses.

### Results

#### Extracting Confounding Factors

The results are shown in Table 2. The analysis of confounding factors revealed a significant association of depressive symptoms with gender and role in the team. However, no association was found for age, grade, competition years, or events.

**TABLE 2:** The relationship between demographic variables and depressive symptoms.

		*n*	%	SDS		*t*-Value/*F*-value	*p*-Value
				Mean	*SD*		
Sex	Male	109	47.0	38.94	7.65	−2.30*^a^*	0.022
	Female	123	53.0	41.36	8.26		
Role in the team	Regular	119	51.3	39.06	7.97	−2.28*^a^*	0.023
	Not regular	113	48.7	41.45	7.99		
Age	≤18 years	48	20.7	40.83	7.42	0.25*^b^*	0.865
	19 years	72	31.0	40.53	7.99		
	20 years	78	33.6	39.81	8.26		
	≥21 years	34	14.7	39.68	8.79		
School year	1st	63	27.2	40.30	7.69	0.39*^b^*	0.760
	2nd	111	47.8	40.11	8.34		
	3rd	37	15.9	39.51	7.08		
	4th	21	9.1	41.86	9.44		
Competitive years	1–5 years	23	9.9	38.09	8.60	1.12*^b^*	0.328
	6–10 years	91	39.2	40.88	7.86		
	≥11 years	118	50.9	40.14	8.09		
Competitive event	Individual sports	80	34.5	39.89	8.09	−0.46*^a^*	0.645
	Team sports	152	65.5	40.40	8.05		

*SDS, Self-Rating Depression Scale (Zung, 1965).*

*^a^t-values, ^b^F-values.*

#### The Relationship Between Organization-Based Self-Esteem and Depression Symptoms

**FIGURE 2:**
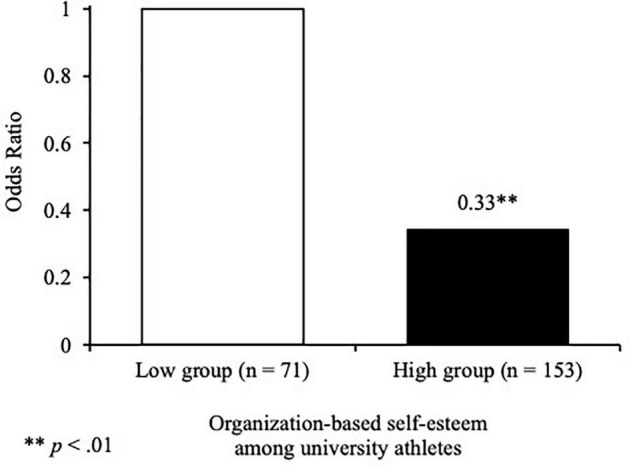
The relationship between organization-based self-esteem and risk of depressive symptoms.

To examine the relationship between organization-based self-esteem and depressive symptoms among university athletes, a binomial logistic regression analysis was performed using the OBSE-UA score as the independent variable, depressive symptoms as dependent variables, and gender and role in the team as confounders. The results showed that the prediction accuracy of this model was guaranteed from the tests of Hosmer and Lemeshow (χ^2^ = 2.95, *df* = 5, *p* > 0.05). A significant relationship [OR (95% CI) = 0.33 (0.15-0.69), *p* < 0.01] was also shown, suggesting that athletes with high organization-based self-esteem were approximately 0.33 times less likely to develop moderate or higher depressive symptoms than athletes with low organization-based self-esteem (Figure 2). Binomial logistic regression analysis showed significant relationships with the following depressive symptoms: “personal devaluation,” “emptiness,” “dissatisfaction,” “psychomotor retardation,” “decreased appetite,” “confusion,” and “indecisiveness” (Table 3).

**TABLE 3:** The relationships between organization-based self-esteem and risk of depressive symptoms.

SDS items	*OR*	*p*-Value	95%CI
Personal devaluation	0.11	<0.001	0.04 – 0.27
Suicidal ideation	0.20	0.180	0.02 – 2.09
Emptiness	0.27	<0.001	0.14 – 0.52
Dissatisfaction	0.33	0.001	0.18 – 0.63
Psychomotor retardation	0.37	0.003	0.19 – 0.70
Depressed affect	0.43	0.053	0.18 – 1.01
Tachycardia	0.47	0.376	0.09 – 2.47
Decreased appetite	0.47	0.047	0.22 – 0.99
Confusion	0.49	0.035	0.26 – 0.95
Indecisiveness	0.49	0.049	0.24 – 1.00
Decreased libido	0.52	0.074	0.26 – 1.06
Hopelessness	0.54	0.053	0.29 – 1.01
Psychomotor agitation	0.58	0.329	0.29 – 1.75
Sleep disturbance	0.59	0.189	0.27 – 1.29
Fatigue	0.85	0.630	0.45 – 1.63
Irritability	1.08	0.885	0.37 – 1.91
Worse in the morning	1.08	0.874	0.43 – 2.73
Crying spells	1.09	0.872	0.38 – 3.18
Weight loss	1.36	0.548	0.50 – 3.67
Constipation	2.22	0.117	0.82 – 5.99

*Items were sorted by ascending order using OR.*

*SDS, Self-Rating Depression Scale (Zung, 1965).*

## Discussion

### Validity and Reliability of the Organization-Based Self-Esteem Scale for University Athletes

In this study, to clarify the relationship between organization-based self-esteem and depressive symptoms among university athletes, we first developed an organization-based self-esteem scale for university athletes (OBSE-UA) in Study I. Results of the exploratory factor analysis for the OBSE-UA showed a one-factor structure similar to that in previous studies dealing with other organization-based self-esteem scales (Pierce et al., 1989; Matsuda et al., 2011). We performed a confirmatory factor analysis to verify the modification indices. We deleted four items from the original 10 items because they were redundant and checked the goodness of the model fit again. The results showed that CFI, GFI, AGFI, TLI were 0.95 or more, and RMSEA and SRMR were less than 0.08 Compared with the criteria of model goodness of fit, we considered that a generally good model was shown (Browne and Cudeck, 1992; Hu and Bentler, 1999).

In a previous study on corporate employees (Pierce et al., 1989), 10 items were used to assess organization-based self-esteem, whereas, in this study on university athletes, there were six items. The four items that we deleted were: “I am trusted in my team,” “There is faith in me in my team,” “I am a valuable member of this team,” and “I am an important member of this team.” These items reflect individuals’ status within the organization (“I am trusted in my team” and “My team has faith in me”) and their evaluation within the organization (“I am a valuable member of this team” and “I am an important member of this team”) were excluded. Whereas the extracted items that are included in the final version of the OBSE-UA pertain to competence demonstrated in the organization (e.g., “I can make a difference in my team” and “I am efficient in my team”). Therefore, it may be appropriate to conclude that, for university athletes, competence demonstrated in the organization is a major element of self-esteem within the organization. This may be due to the competitive environment that athletes live in. For example, university athletes who play individual sports may believe that they can contribute to the team by performing better on their own than by finding value in the evaluation of others. In Study I, 108 participants were involved in individual sports, while 102 were involved team sports; thus, it is possible that items pertaining to status and evaluations by others were omitted due to differences in the athletes’ competition environment.

Furthermore, statistically sufficient results were obtained to confirm the reliability of the OBSE-UA, as in previous studies on organization-based self-esteem scales (Pierce et al., 1989; Matsuda et al., 2011). In this study, the reproducibility was examined for 2 weeks, and it was suggested that the item average of the OBSE-UA was within the fluctuation of about 0.1 points. The 95% CI of limit of agreement in fluctuation was ±0.95 (lower −0.95 to 0.95) points. From this value, if over 95% CI of limit of agreement in fluctuation (±0.95) of this scale by some intervention is found, it can be regarded as the intervention effect. The internal consistency was 0.89, which corresponds to the criterion of almost perfect (Landis and Koch, 1977). From these results, it can be concluded that the organization-based self-esteem scale was created to measure the self-esteem of university athletes belonging to various sports teams.

### The Relationship Between Organization-Based Self-Esteem and Risk of Depressive Symptoms

This study investigated the relationship between organization-based self-esteem and risk of depressive symptoms among university athletes using the newly developed OBSE-UA. The results showed that athletes with high organization-based self-esteem were approximately 0.33 time less likely to develop moderate or severe depressive symptoms than athletes with low organization-based self-esteem. In other words, athletes who feel they have a significant presence and role in their team are less likely to develop severe depressive symptoms.

This study is novel in that it clarified, for the first time, the relationship between university athletes’ organization-based self-esteem and risk of depressive symptoms and confirmed the effect by odds ratios. When targeting employees and workers, it has been reported that those with high self-esteem within the organization are less likely to exhibit depressive symptoms (Jex and Elacqua, 1999; Bowling et al., 2010; Matsuda et al., 2011). Additionally, these findings validated this study’s hypothesis about the association between organization-based self-esteem and depressive symptoms among athletes and Kuettel and Larsen’s (2020) notion that athletes’ perception of their competence within their team is a protective factor against depressive symptoms. Hence, increasing organization-based self-esteem among athlete may help reduce depressive symptoms. Nevertheless, as these studies used linear regression analyses, the relevance of their results was clarified, but not the risks. Therefore, it is suggested that athletes are provided coaching which is aimed at improving and preventing depressive symptoms among them. Additionally, researchers should consider conducting longitudinal studies to investigate the causal effect relationship between organization-based self-esteem and risk of depressive symptoms among athletes.

In Study II, we examined the relationship between organization-based self-esteem and risk of depressive symptoms measured by the SDS. Results showed that athletes with high organization-based self-esteem have a lower risk of experiencing “personal devaluation,” “emptiness,” “dissatisfaction,” “psychomotor retardation,” “decreased appetite,” “confusion,” and “indecisiveness” than athletes with low organization-based self-esteem. These symptoms correspond mainly to cognitive and somatic symptoms of depression. Kitamura et al. (2004) conducted a factor analysis on each symptom in the SDS in university students and found that the SDS contains three factors: affective, cognitive, and somatic. In this study, “personal devaluation,” “emptiness,” “dissatisfaction,” and “indecisiveness” which were shown to be related to self-esteem within the organization, were included among the affective factors. “Psychomotor retardation,” “decreased appetite,” and “confusion” were included among somatic factors. This suggests that organization-based self-esteem in university athletes may reduce the risk of developing symptoms related to the cognitive and somatic aspect of depression. Whereas there was almost no association between the affective symptoms of depression and organization-based self-esteem. Therefore, organization-based self-esteem may not be involved in the affective symptoms of depression. As organization-based self-esteem seems to be associated with the cognitive and somatic aspects of depression, it may be possible to mitigate relatively minor depressive symptoms by promoting high organization-based self-esteem. However, it may be difficult to mitigate depressive symptoms at a pathological level of the emotional and physical aspects. As it is expected that the cognitive and somatic aspects of depressive symptoms are also significantly involved in the performance of athletes, it is important for athletes to successfully develop self-esteem within the organization to enhance performance.

Based on the above, it is necessary to examine factors that enhance the self-esteem of athletes within the organization for a healthy competitive life. Studies on corporate employees have shown that organizational support is involved in maintaining organization-based self-esteem. Specifically, employees who felt that they enjoyed support from the organization were shown to have high self-esteem within the organization (Ferris et al., 2009; Matsuda et al., 2011). Moreover, employees who rated the leaders of their organization as humble also had high self-esteem within the organization (Zhang and Liu, 2019). Therefore, assuming a sports instruction scene, social support from teammates, and supportive leadership behavior by instructors may be crucial factors to promote organization-based self-esteem among athletes. These conclusions need to be verified in future research, using the scale developed in this study.

Finally, the limitations of the study and future issues are described. First, the outcome of this study was limited to depressive symptoms. However, athletes face diverse mental health problems, and depressive symptoms are only one of the responses to stress observed in athletes. Therefore, it is necessary to examine the relationship between organization-based self-esteem and mental health problems apart from depressive symptoms. Second, organization-based self-esteem may be involved in cognitive stressors. High self-esteem within the organization was not found to be associated with job stressors (ambiguity, conflicts, and overload in roles), but this association was observed in those with low self-esteem within the organization (Pierce et al., 1993). Job stressors may reduce job satisfaction (Pierce et al., 1993). Therefore, in the future, it is necessary to examine the relationship between self-esteem within the organization and stress in athletes. Third, the current study had a cross-sectional research design. To clarify the causal relationship between organization-based self-esteem and risk of depressive symptoms, it is necessary to design a longitudinal study to examine how organization-based self-esteem is involved in the development of depressive symptoms.

## Conclusion

This study developed a scale with sufficient reliability and validity to assess organization-based self-esteem in university athletes. Athletes with high organization-based self-esteem were 0.33 time less likely to develop depressive symptoms than athletes with low organization-based self-esteem. Thus, facilitating athletes’ acquisition of organization-based self-esteem may play an important role in preventing depressive symptoms among university athletes. In addition, organization-based self-esteem in university athletes can be measured using the OBSE-UA developed in this study. The development of this scale will promote research activities regarding organization-based self-esteem in university athletes.

## Data Availability Statement

The raw data supporting the conclusions of this article will be made available by the authors, without undue reservation.

## Ethics Statement

The studies involving human participants were reviewed and approved by the Research Ethic Committee of Juntendo University. The patients/participants provided their written informed consent to participate in this study. Written informed consent was obtained from the individual(s) for the publication of any potentially identifiable images or data included in this article.

## Author Contributions

RN designed the study, collected all the data, performed the statistical analysis, and prepared the manuscript. YK supported all processes of the study (study design, data collection, statistical analysis, and manuscript preparation). Other authors provided expert comments for the scale development according to their specialties (SY: health psychology; NS and TO: psychiatry). All authors read and approved the final manuscript.

## Conflict of Interest

The authors declare that the research was conducted in the absence of any commercial or financial relationships that could be construed as a potential conflict of interest.

## Publisher’s Note

All claims expressed in this article are solely those of the authors and do not necessarily represent those of their affiliated organizations, or those of the publisher, the editors and the reviewers. Any product that may be evaluated in this article, or claim that may be made by its manufacturer, is not guaranteed or endorsed by the publisher.
